# Deleting the mouse *Hsd17b1* gene results in a hypomorphic *Naglu* allele and a phenotype mimicking a lysosomal storage disease

**DOI:** 10.1038/s41598-017-16618-5

**Published:** 2017-11-27

**Authors:** Heli Jokela, Janne Hakkarainen, Laura Kätkänaho, Pirjo Pakarinen, Suvi T. Ruohonen, Manuel Tena-Sempere, Fu-Ping Zhang, Matti Poutanen

**Affiliations:** 1 Research Centre for Integrative Physiology and Pharmacology, Institute of Biomedicine and Turku Center for Disease Modeling, University of Turku, FI-20014 Turku, Finland; 2 Department of Cell Biology, Physiology and Immunology, and Instituto Maimónides de Investigación Biomédica de Córdoba & Hospital Universitario Reina Sofia (IMIBIC/HURS), ES-14004 Cordoba, Spain; 3 Centre for Bone and Arthritis Research at Institute of Medicine, Sahlgrenska Academy at University of Gothenburg, SE-41345 Gothenburg, Sweden

## Abstract

HSD17B1 is a steroid metabolising enzyme. We have previously generated knockout mice that had the entire coding region of *Hsd17b1* replaced with *lacZ-neo* cassette (Hsd17b1-LacZ/Neo mice). This resulted in a 90% reduction of HSD17B1 activity, associated with severe subfertility in the knockout females. The present study indicates that Hsd17b1-LacZ/Neo male mice have a metabolic phenotype, including reduced adipose mass, increased lean mass and lipid accumulation in the liver. During the characterisation of this metabolic phenotype, it became evident that the expression of the *Naglu* gene, located closely upstream of *Hsd17b1*, was severely reduced in all tissues analysed. Similar results were obtained from Hsd17b1-LacZ mice after removing the *neo* cassette from the locus or by crossing the Hsd17b1-LacZ/Neo mice with transgenic mice constitutively expressing human *HSD17B1*. The deficiency of *Naglu* caused the accumulation of glycosaminoglycans in all studied mouse models lacking the *Hsd17b1* gene. The metabolic phenotypes of the *Hsd17b1* knockout mouse models were recapitulated in *Naglu* knockout mice. Based on the data we propose that the *Hsd17b1* gene includes a regulatory element controlling *Naglu* expression and the metabolic phenotype in mice lacking the *Hsd17b1* genomic region is caused by the reduced expression of *Naglu* rather than the lack of *Hsd17b1*.

## Introduction

The ES cells used in the present study were generated by using a method based on BAC recombineering technology (VelociGene). The strategy replaced the entire open reading frame between the translation initiation codon (ATG) and the stop codon of the *Hsd17b1* gene with the *lacZ-neo* cassette. The technique is efficient in generating large-sized deletions and inserting reporter genes into the translation initiation site and has been applied to generate targeted alleles in a high-throughput manner^1^. Targeting vectors, ES cells targeted by the method, and a collection of mouse models generated with the vectors are available for the scientific community by The International Mouse Phenotyping Consortium (http://www.mousephenotype.org/)^2^.

Deleting large regions of genomic sequence or inserting large DNA fragments might also result in unintended consequences on the regulation of adjacent and even distant transcriptional units, such as a severe downregulation of neighbouring genes^3–5^. When the classical pronuclear injection technique is used for transgenic mouse production, the potential integration site effects on transgene expression or in the expression of other genes are typically controlled by generating several independent mouse lines^6^. However, in gene targeting studies, several mouse lines with different mutation strategies are typically not generated. Nevertheless, the potential impact of the gene targeting on the chromatin structure and on the expression of genes around the targeted locus has recently gained more interest. The recent results indicate the importance of considering the effect of the mutation on the expression of closely located flanking genes^5^. These effects might well be one of the reasons for the observed differences in various knockout (KO) mouse models generated for the same gene.

HSD17B1, the enzyme studied in the present report, belongs to hydroxysteroid (17-beta) dehydrogenases (HSD17Bs), which catalyse the conversion of 17-ketosteroids, such as oestrone (E1) and androstenedione (Adione), into the corresponding 17-hydroxysteroids, oestradiol (E2) and testosterone (T). The HSD17B activity is essential for the formation of the highly active circulating sex steroids in the gonads. However, HSD17B activity is also present in the peripheral target tissues of sex steroid action^7^, thereby regulating the local intra-tissue sex steroid concentrations^8,9^. The HSD17B family contains 14 members, HSD17B1-14, and each of them has a distinctive expression profile and physiological function^7,10^. Mouse *Hsd17b1* is mainly expressed in the ovaries, while expression can also be detected in the testis and white adipose tissue^11–13^. In line with the expression profile, we have recently demonstrated that HSD17B1 activity has a critical role in ovarian steroid synthesis and fertility in female mice^13^. The enzyme has been shown to be responsible for 90% of the total HSD17B activity in mouse ovaries and the Hsd17b1-LacZ/Neo females are severely subfertile. In addition to the identified changes in ovarian function, the phenotyping analyses carried out by the International Mouse Phenotyping Consortium (http://www.mousephenotype.org/) associated the *Hsd17b1* gene deletion in the Hsd17b1-LacZ/Neo mice with altered body composition, immune system phenotype and abnormal behaviour.

In the present study, we aimed to further define the potential role of HSD17B1 in phenotypic changes in non-reproductive tissues using the Hsd17b1-LacZ/Neo mice. Our data revealed that the deletion of the *Hsd17b1* gene and its replacement with *lacZ* markedly reduced the expression of the immediate upstream gene, N-acetyl-alpha-glucosaminidase (*Naglu*). Our extensive characterisation of the metabolic phenotype of the mouse models that present with various genetic modifications at the *Hsd17b1* and *Naglu* alleles indicates that the effects observed in the body composition and liver function in the *Hsd17b1* knockout models are caused by a reduced expression of *Naglu* and are not due to a deficiency in HSD17B1 enzyme activity.

## Results

### Mouse *Hsd17b1* gene includes a genomic region regulating *Naglu* gene expression

**Figure 1:**
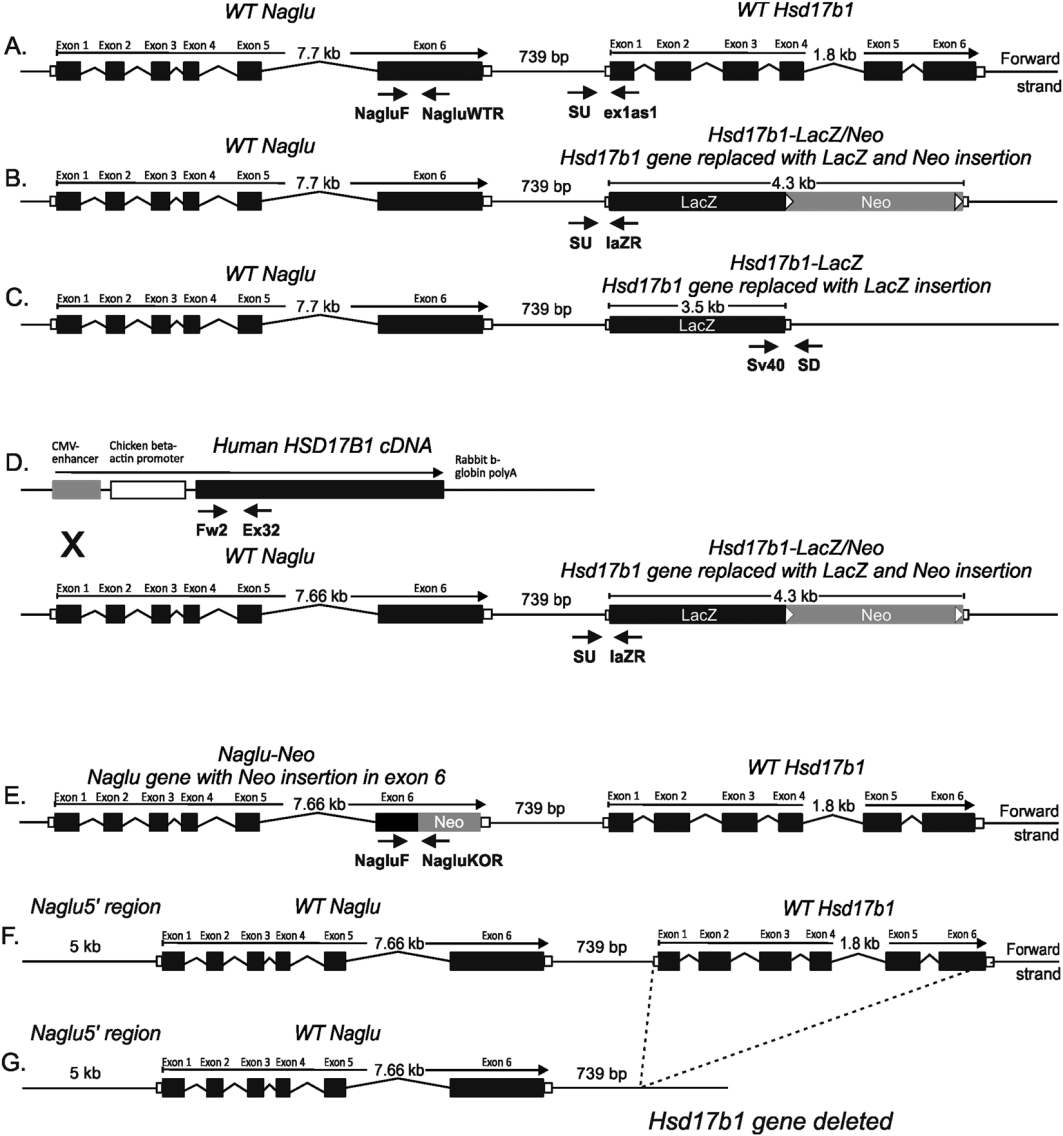
The schematic diagram of the alleles of mouse models used in this study. (**A**) Structure of the wild type (WT) locus of the *Naglu* and *Hsd17b1* genes showing 6 exons presented as black boxes for both the *Naglu* and *Hsd17b1* genes. (**B**) The targeted allele in Hsd17b1-LacZ/Neo mice with a deleted *Hsd17b1* gene, including the targeting vector containing the *lacZ* and *neo* cassette^13^. (**C**) The targeted allele in Hsd17b1-LacZ mice, produced by crossing the Hsd17b1-LacZ/Neo mice with the Rosa26 transgenic mouse strain^14^. (**D**) Transgenic mice ubiquitously expressing the human *HSD17B1* gene^16^ were crossed with Hsd17b1-LacZ/Neo mice to bring the HSD17B1 enzyme activity to an unrelated locus. In the HSD17B1 transgenic mice, human *HSD17B1* cDNA was inserted between the CMV-enhanced chicken beta-actin promoter and rabbit beta-globin poly-A tail. (**E**) The targeted allele in Naglu-Neo mice^15^, showing a *neo* cassette inserted into exon 6. Arrows indicate the genotyping primer sites for all mouse models. (**F**) The interaction of the *Hsd17b1* and *Naglu* gene region was also studied *in vitro* using expression constructs including a genomic fragment extending from a region 5 kb upstream of the transcription start site of *Naglu* until the 3′-region of the *Hsd17b1* gene, and using a similar construct of which we deleted 1.8 kb long fragment of the *Hsd17b1* gene.

**Figure 2:**
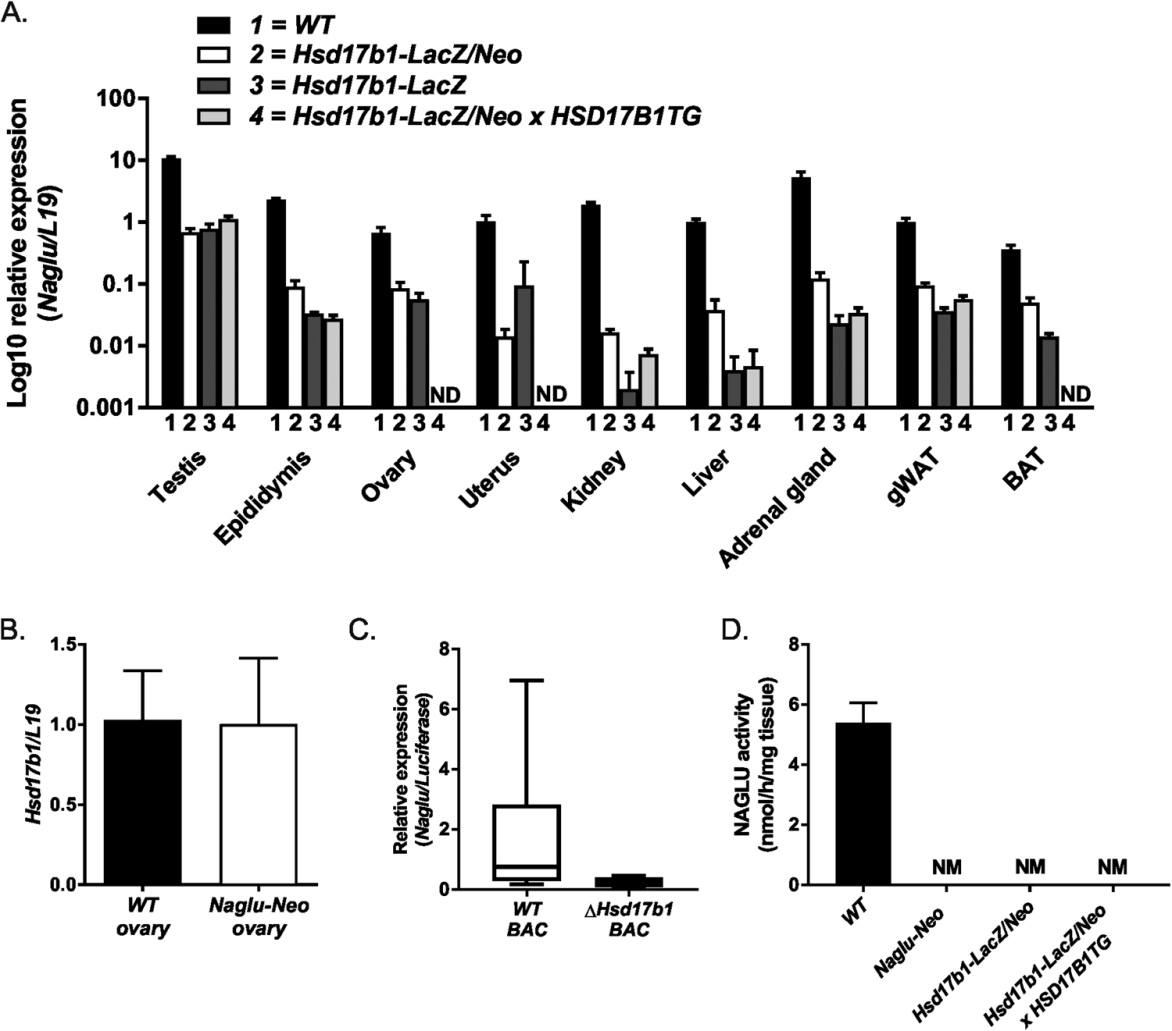
Expression analysis of *Naglu* and *Hsd17b1*. Quantitative RT-PCR (**A**) analysis of the testis, epididymis, ovary, uterus, kidney, liver, adrenal gland, gonadal white adipose tissue (gWAT) and brown adipose tissue (BAT) showed the downregulation of *Naglu* expression in the Hsd17b1-LacZ/Neo mice (white bars), Hsd17b1-LacZ mice (dark grey bars) and Hsd17b1-LacZ/Neo X HSD17B1TG mice (light grey bars) compared with WT mice (black bars) at 3 months of age. (**B**) The *Hsd17b1* mRNA expression was not altered in Naglu-Neo mice ovaries. Bars demonstrate the mean of the relative expression normalised to the expression of *L19* (the error bars show SD, n = 6). (**C**) The expression of *Naglu* was downregulated in the BAC vector with the entire *Hsd17b1* gene deleted (expression normalised to *luciferase* gene expression and error bars show SEM, n = 6). (**D**) Naglu activity was proven to be disrupted in the livers of Naglu-Neo, Hsd17b1-LacZ/Neo and Hsd17b1-LacZ/Neo X HSD17B1TG mice. Bars demonstrate the mean activity (Error bars SEM, n = 3–5). ND = not determined, NM = not measurable.

We recently generated *Hsd17b1* knockout mice by using the C57Bl/6N background ES cells obtained from the KOMP Repository^13^. In these targeted ES cells, the coding region of the *Hsd17b1* gene was replaced with a sequence that included a *lacZ* reporter gene, which was inserted in the translation start site of the *Hsd17b1* locus, followed by a *neo* cassette (Fig. 1B, Hsd17b1-LacZ/Neo mice). In mice, *Hsd17b1* is highly expressed in the ovary. Accordingly, our recent results demonstrated that the Hsd17b1-LacZ/Neo mice presented with altered ovarian function, associated with the expected reduced level of HSD17B1 activity and the increased ratio of 17-keto steroids to 17-beta-hydroxysteroids^13^. However, the RNA sequencing data of two different tissues indicate that in the Hsd17b1-LacZ/Neo mice, the mRNA expression of immediate 5′ gene, N-acetyl-alpha-glucosaminidase (*Naglu*, located 8.3-0.7 kb upstream of the *Hsd17b1* transcription start site), was severely reduced. This was confirmed by the qRT-PCR data indicating a marked decrease in *Naglu* mRNA in both the male and female tissues (7- to 119-fold, Fig. 2A and Supplementary Figure S1A). The downregulation was gene-specific, as the RNAseq data obtained from the ovary and male adipose tissue showed that the expression of other genes located in the vicinity of *Hsd17b1* (500 kb upstream and downstream) were not similarly altered (Supplementary Table S1).

Due to the potential effect of the human ubiquitin C (*UBC*) promoter, present as part of the inserted *neo* cassette, we deleted the *neo* expressing cassette from the targeted *Hsd17b1* locus by crossing the Hsd17b1-LacZ/Neo mice with those expressing *Cre* recombinase under the *Rosa26* promoter^14^. In these mice (Hsd17b1-LacZ) the *neo* cassette was removed in the germ line, resulting in a lack of the selection cassette in all cells (Fig. 1C). However, the qRT-PCR results (Fig. 2A and Supplementary Figure S1A) indicated that in both males and females the *Naglu* mRNA expression was still downregulated in a similar fashion as was observed in the Hsd17b1-LacZ/Neo mice (11- to 228-fold). Thus, the presence of the *neo* cassette in the targeted locus was not the cause of the reduced expression of *Naglu*. The modest difference in the *Naglu* expression between the different *Hsd17b1* knockout mouse lines is likely due to the different genetic backgrounds. We also sequenced the genomic region between the beginning of exon 6 of the *Naglu* gene and the *Hsd17b1* transcription start site, as well as the 3′ end of the *lacZ-neo* cassette insertion site, and confirmed the proper targeting without the presence of any deletions or mutations at these regions. We next studied the *Hsd17b1* expression in mice with the disrupted *Naglu* gene generated by an insertion of a *neo* cassette within exon 6 (Naglu-Neo mice)^15^. The data demonstrated that this insertion did not have an effect on the expression of *Hsd17b1* (Fig. 2B). To further prove the interaction between the *Hsd17b1* and *Naglu* loci, we generated two expression constructs for *in vitro* studies. One of the constructs extended from a site 5 kb upstream of the *Naglu* transcription start site until the 3′ region of the *Hsd17b1* gene (Fig. 1F), and the other one was similar but lacked the 1.8 kb long coding region of the *Hsd17b1* gene (Fig. 1G). By transfecting these constructs into COS cells, it was evident that significant transcription of *Naglu* was observed only with the construct including the *Hsd17b1* gene region (Fig. 2C). Thus, both *in vivo* and *in vitro* data indicate the presence of an important regulatory region within the *Hsd17b1* gene controlling *Naglu* expression. The *Hsd17b1* gene deletion resulted in a hypomorphic *Naglu* allele and to undetectable level of NAGLU activity in the liver (Fig. 2D). In contrast, the disruption of the last exon of the *Naglu* gene did not interfere with *Hsd17b1* expression (Fig. 2B), while the NAGLU activity was abolished (Fig. 2D).

### Downregulation of *Naglu* in the Hsd17b1-LacZ/Neo mice results in an increase in cellular glycosaminoglycan and lysosomal accumulation

**Figure 3:**
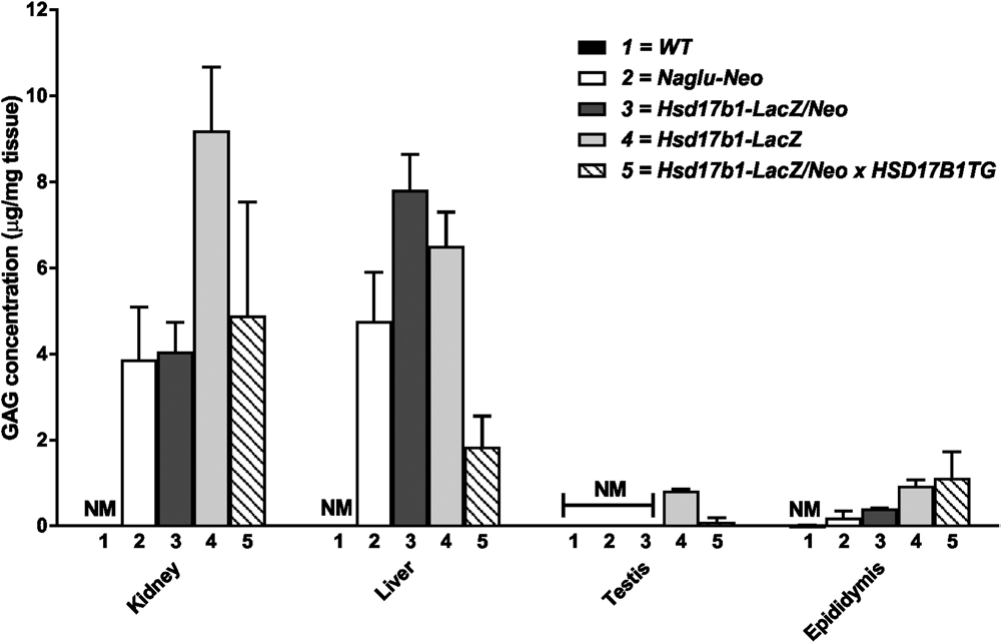
Measurement of soluble GAG in the analysed tissues. Alcian blue precipitable GAG (mean ± SEM) was analysed from tissues of Naglu-Neo, Hsd17b1-LacZ/Neo, Hsd17b1-LacZ and Hsd17b1-LacZ/Neo X HSD17B1TG mice and their control littermates (n = 3). NM = not measurable.

The *Naglu* gene codes for an enzyme that is involved in the catabolism of glycosaminoglycans (GAGs) by hydrolysing the terminal N-acetyl-D-glucosamine residues in N-acetyl-alpha-D-glucosaminides. Due to the reduced enzymatic activity, the accumulation of GAGs in various male and female organs was identified in the Hsd17b1-LacZ/Neo mice, similar to that measured in the Naglu-Neo mice. The highest GAG concentrations were measured in male and female liver and kidney (Fig. 3 and Supplementary Figure S1B), where the difference between the Hsd17b1-LacZ/Neo and WT mice was 2.5- and 7.8-fold. A more modest difference in GAG concentration between the WT and Hsd17b1-LacZ/Neo mice was observed in the ovary (Supplementary Figure S1B). Interestingly, in the testis with the highest *Naglu* expression in the WT mice, the remaining NAGLU activity present in the Hsd17b1-LacZ/Neo mice (10% from the WT mice) was enough to avoid the accumulation of GAG. To further assess the contribution of *Hsd17b1* ablation to this GAG-accumulation, we overexpressed the human HSD17B1 enzyme in our null mouse model. As a result, very similar GAG accumulation was observed in Hsd17b1-LacZ and Hsd17b1-LacZ/Neo X HSD17B1TG mice. Thus, deleting the *neo* cassette or introducing HSD17B1 activity by a transgene (shown to result in increased HSD17B activity in previous studies^16^, and confirmed in the present study, Supplementary Figure S2) did not restore *Naglu* activity in mice with the disrupted *Hsd17b1* gene.

**Figure 4:**
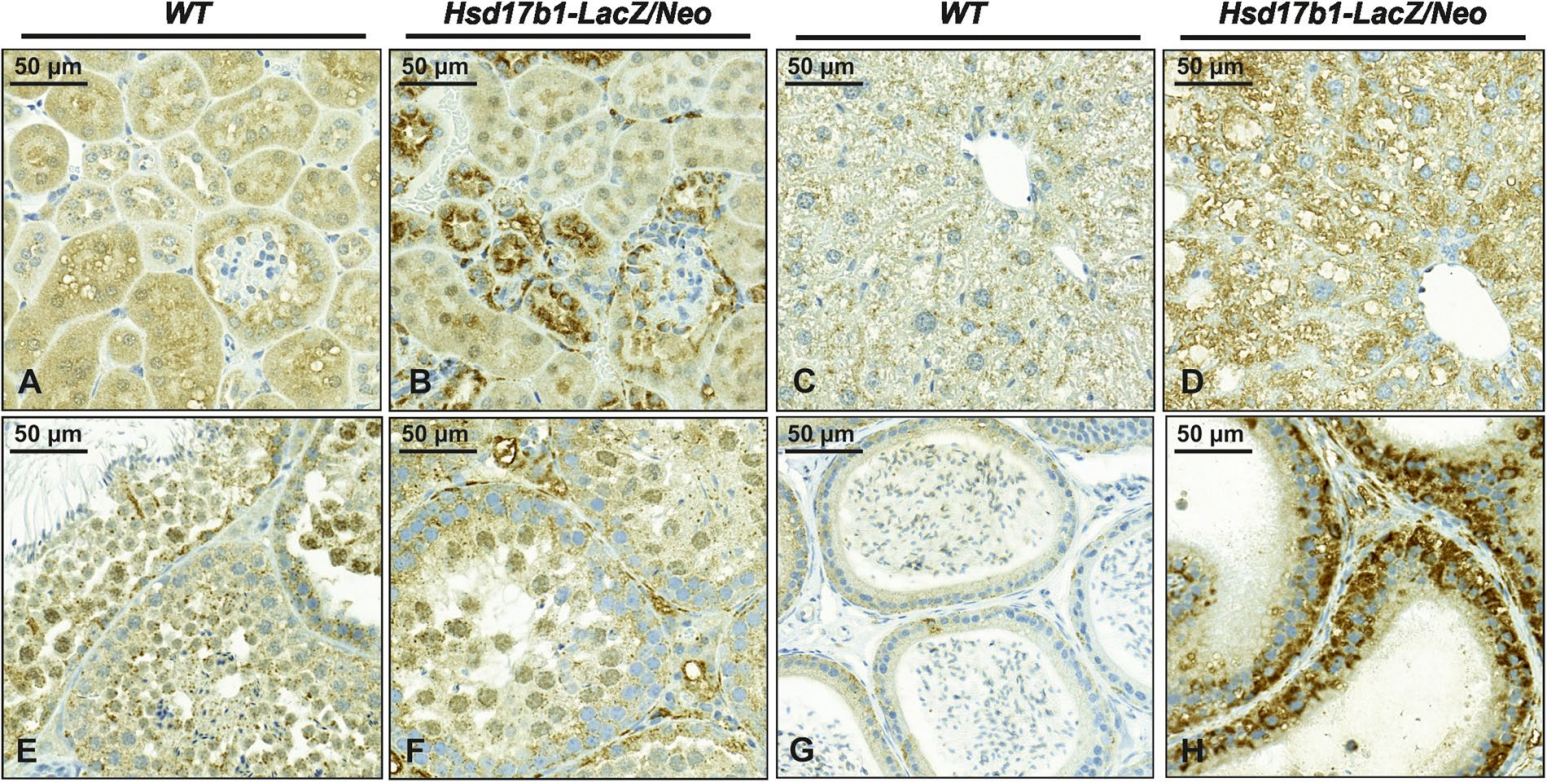
Immunohistochemical analysis of Naglu-Neo and Hsd17b1-LacZ/Neo male mice. Immunohistochemical staining for LAMP1 was used to demonstrate lysosomes in the Hsd17b1-LacZ/Neo mice. Increased staining was observed in the kidney (**B**), liver (**D**) and epididymis (**H**) compared to the WT (**A**,**C** and **G**), while no difference in the intensity of staining was shown in testis sections from the WT and Hsd17b1-LacZ/Neo mice (**E** and **F**).

The increased amount of GAGs within the tissues of Hsd17b1-LacZ/Neo mice coincided with the increased signal intensity for a lysosomal marker protein (LAMP1) measured by immunohistochemical staining. The highest staining intensity was observed in the kidneys of males (Fig. 4B), epididymis (Fig. 4H) and uterus (Supplementary Figure S1D), and moderate staining was seen in the male liver (Fig. 4D) and ovary (Supplementary Figure S1F), while no difference in staining was observed in the testicular sections from WT and Hsd17b1-LacZ/Neo mice (Fig. 4E,F). The signal for LAMP1 immunohistochemistry co-localized well with the areas of large vacuoles present in the haematoxylin and eosin (HE) stained histological sections from the Hsd17b1-LacZ/Neo mouse uterus (Supplementary Figure S1H) and ovary (Supplementary Figure S1J). Similar results were obtained with another lysosomal marker, LIMP2, except that intense staining was also observed in the stromal vacuoles of the ovary (Supplementary Figure S3). Histological staining also confirmed the appearance of these vacuoles in the Naglu-Neo mice (Supplementary Figure S1L,N). Thus, the vacuolated phenotype of the female reproductive tissues is most likely due to the accumulation of GAGs caused by the lack of proper expression of *Naglu*.

Although similar accumulation of GAGs was detected in the ovaries of Naglu-Neo mice and mice with a targeted disruption of the *Hsd17b1* gene, the Naglu-Neo mice did not present with a severe subfertility, as was recently observed for the Hsd17b1-LacZ/Neo mice^13^. The Naglu-Neo females showed a regular estrous cycle and produced a similar litter size compared to the WT females, and did not display alterations in the expression of the steroidogenic genes (Supplementary Figure S4).

### Metabolic phenotype of the Hsd17b1-LacZ/Neo male mice is associated with the reduced expression of *Naglu*

**Figure 5:**
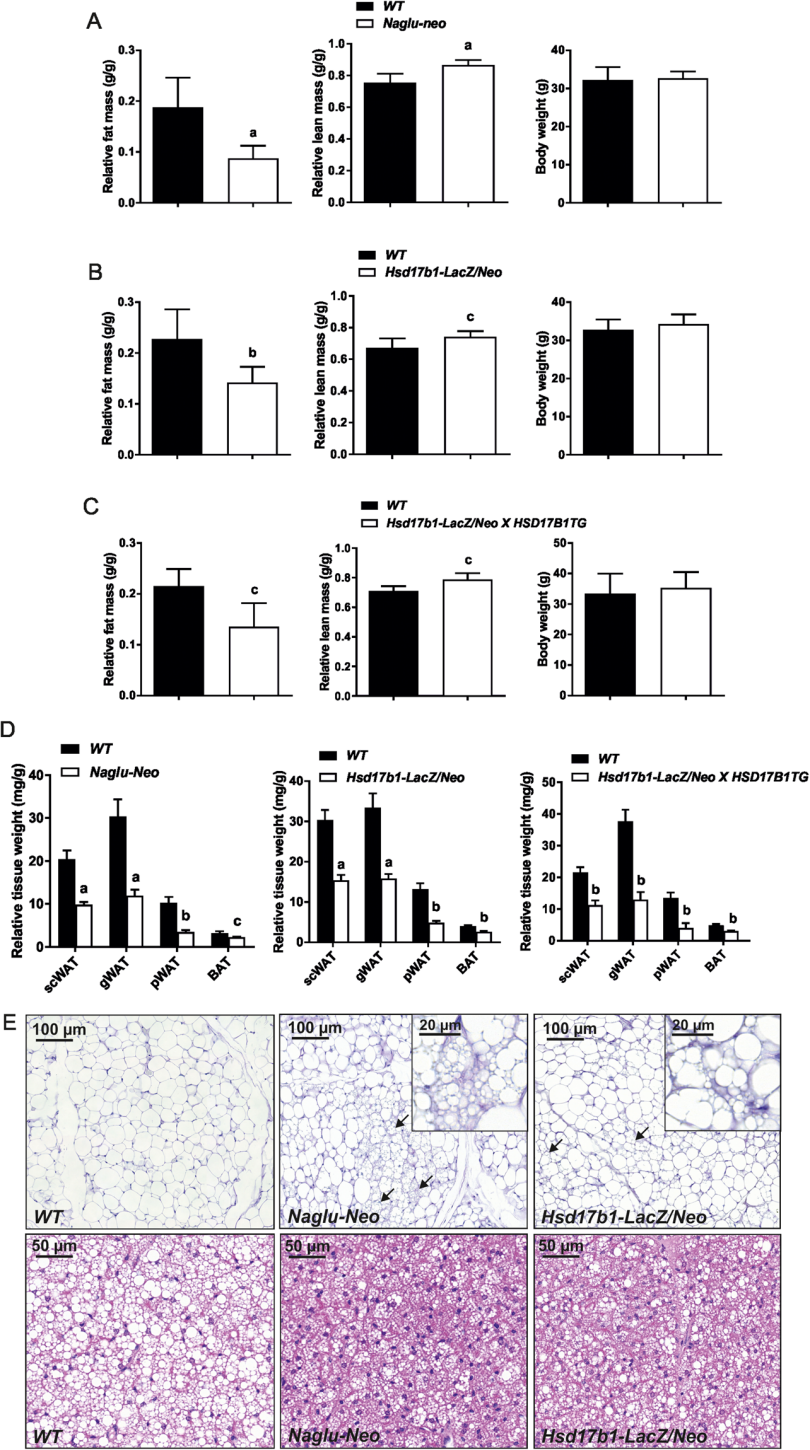
Measurement of the fat and lean mass in the Naglu-Neo, Hsd17b1-LacZ/Neo and Hsd17b1-LacZ/Neo X HSD17B1TG male mice. (**A**) The fat mass was markedly reduced, and the lean mass was significantly increased in the Naglu-Neo mice (n = 9–10), while the body weight was not changed. Similar alterations were also observed in the Hsd17b1-LacZ/Neo (n = 10–11) (**B**) and Hsd17b1-LacZ/Neo X HSD17B1TG mice (n = 5–6) (**C**). (**D**) The weight of all the white adipose depots (scWAT = subcutaneous white adipose tissue, gWAT = gonadal white adipose tissue and pWAT = perirenal white adipose tissue), along with the brown adipose tissue (BAT), were decreased significantly. Statistical significance is marked with letters indicating P values: a, p < 0.001 compared to WT; b, p < 0.01 compared to WT; c, p < 0.05 compared to WT. Bars show mean value and error bars show SD. (E) Histological analysis showed smaller adipocytes in the WAT and the lipid droplets in BAT were smaller in size. Furthermore, the subcutaneous WAT of the Naglu-Neo and Hsd17b1-LacZ/Neo mice showed signs of browning (arrows), which is shown more detailed in insertions.

**Figure 6:**
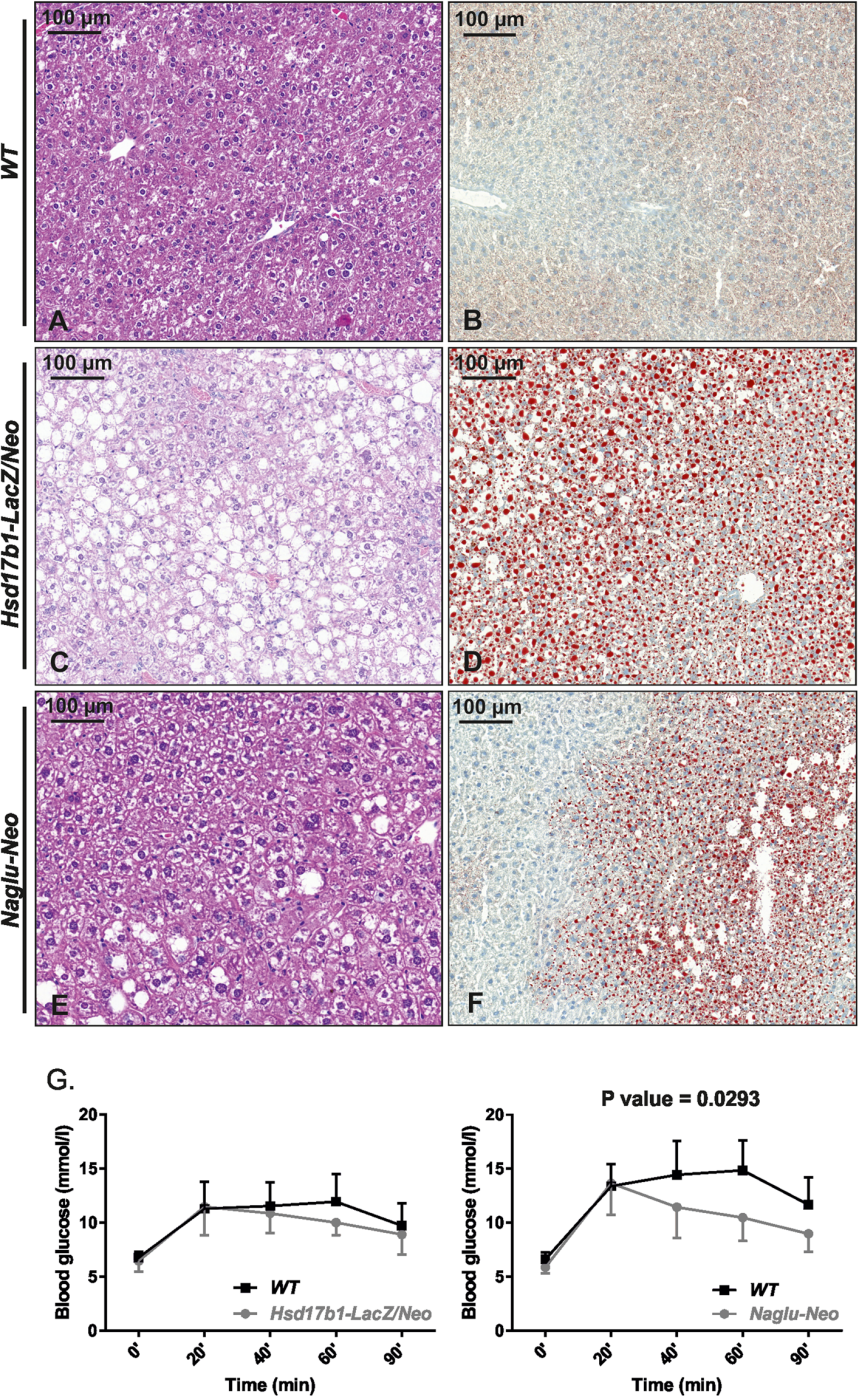
Liver histology and glucose tolerance test (GTT). When compared to the WT (**A** and **B**), the histology showed that both the Hsd17b1-LacZ/Neo and Naglu-Neo mice had lipid accumulation in the liver, as indicated by HE staining (**C** and **E**) and by Oil Red O staining (**D** and **F**). Glucose tolerance was improved in the Naglu-Neo mice (n = 9–10) despite the hepatic lipid accumulation (**G**). The P value is 0.0293. Error bars show SD.

It has been previously shown that decreased adiposity is a universal feature of lysosomal storage diseases^17^. Thus, we characterised the body weight and composition in the Naglu-Neo, Hsd17b1-LacZ/Neo, and Hsd17b1-LacZ/Neo X HSD17B1TG mice. Compared with the WT mice, the fat mass was markedly reduced and the lean mass was significantly increased in Naglu-Neo males, while the body weight was not altered (Fig. 5A). Similar alterations were also observed in Hsd17b1-LacZ/Neo (Fig. 5B) and Hsd17b1-LacZ/Neo X HSD17B1TG (Fig. 5C) mice. Furthermore, in all the mouse models a reduction in the fat mass corresponded to a reduced weight of all the fat pads analysed, and the amount of interscapular brown adipose tissue was also reduced (Fig. 5D). Histological examination of the subcutaneous white and brown adipose tissue revealed smaller adipocyte size and browning of the white adipose tissue of the Naglu-Neo and Hsd17b1/LacZ/Neo males (Fig. 5E). The weight of the liver was significantly increased in both the Naglu-Neo (WT 1488 mg ± 159 mg vs Naglu-Neo 1956 mg ± 286 mg, P value < 0.001) and Hsd17b1-LacZ/Neo (WT 1692 mg ± 196 mg vs Hsd17b1-LacZ/Neo 2547 ± 493 mg, P value < 0.001) mice. In line with this, histological analysis revealed an increased lipid accumulation in the liver of the Hsd17b1-LacZ/Neo (Fig. 6C) and Naglu-Neo (Fig. 6E) mice. The observed lipid accumulation was seen throughout the liver in the Hsd17b1-LacZ/Neo mice, while in the liver of the Naglu-Neo mice, the lipid accumulation was observed mainly in the centrilobular zone, as demonstrated by Oil Red O staining of both the Hsd17b1-LacZ/Neo (Fig. 6D) and Naglu-Neo (Fig. 6F) mice. Despite the lipid accumulation in the liver, glucose tolerance was improved especially in Naglu-Neo mice (Fig. 6G), while the mechanism for this remains to be explored.

## Discussion

It is widely recognised that the targeted disruption of the genome may affect the function of the neighbouring genes, even though only a limited number of reports exist. These off-target effects may hamper the conclusions made about the causality of the changes observed. One of the concerns has been that the expression vector used to drive the antibiotic selection marker during gene targeting could affect the expression of other genes located close to the insertion site^18,19^. Furthermore, the targeting event may disrupt the regulatory and insulating elements, thereby effecting flanking genes^3,20,21^. The present study revealed that deleting the entire 1.8 kb long *Hsd17b1* gene from mice affects the transcription of the neighbouring 5′ gene coding for NAGLU. Consequently, severe downregulation of the *Naglu* expression was observed in all the Hsd17b1-LacZ/Neo tissues analysed. The end of the *Naglu* gene is located just 0.7 kb upstream from the transcription start site of the targeted gene. Therefore, we sequenced the critical parts of the targeted locus and confirmed the proper sequence between exon 6 of the *Naglu* gene and the transcription start site of *Hsd17b1*. Additionally, the sequencing confirmed that the 3′ end of the targeting construct showed a proper targeting event and the genomic region was clear of deletions or mutations.

There are many intragenic regulatory elements found in introns and in the 3′ and 5′ UTRs of genes regulating transcription. These elements have been shown to include secondary promoters, enhancer and repressor elements, and sequences coding for miRNAs^22^. Consequently, there is a risk for an unintended disruption of the genomic region coding for miRNA sequences during gene targeting, *e*.*g*. many miRNAs are encoded by regions located in the introns and exons of the protein coding genes^23^. Furthermore, according to the WashU Epigenome Browser (http://epigenomegateway.wustl.edu/browser/), the mouse *Hsd17b1* gene contains three short interspersed nuclear elements (SINEs). These nuclear elements are believed to be integrated into a complex regulatory network capable of modifying gene expression across the eukaryotic genome, *e*.*g*., by affecting the 3D structure of DNA or by directly interacting with transcriptional repressors and activators^24,25^.

In addition, it is recognised that the insertion of a *neo* cassette, coding for the selection gene used in the targeting, can influence the regulation of neighbouring genes in the mouse^18,26^. However, in the case of the *Hsd17b1* locus, removing the *UBC-neo* cassette from the targeted locus did not restore the expression of the *Naglu* gene. Neither did the insertion of the *neo* cassette in the last exon of the *Naglu* gene (in the Naglu-Neo mice) disrupt the transcription of the downstream *Hsd17b1* gene. We further confirmed that the lack of HSD17B activity was not the cause of reduced *Naglu* expression by expressing the enzyme in the Hsd17b1-LacZ/Neo mice by a transgene inserted in an unrelated genomic region. Thus, we conclude that the physical disruption of the genomic region is the cause for the downregulation of *Naglu*.

In line with the known enzymatic activity of HSD17B1, the Hsd17b1-LacZ/Neo mice presented with the increased ratio of 17-keto steroids over 17-beta-hydroxysteroids in the ovary and with severe subfertility, indicating a critical role for the enzyme in female reproduction^13^. In addition, as shown in the present study, the Hsd17b1-LacZ/Neo male mice presented with reduced adiposity, increased lean mass and liver steatosis. Similar to the results obtained by us, decreased fat mass in the Hsd17b1-LacZ/Neo mice has been observed by work carried out in the IMPC (http://www.mousephenotype.org/). In contrast to our data, the IMPC analyses showed decreased lean mass in the Hsd17b1-LacZ/Neo mice. However, our data clearly indicate that the targeting method used to delete the *Hsd17b1* gene also results in a marked downregulation of *Naglu* expression. The NAGLU enzyme is involved in the degradation of heparan sulfate, and reduced expression or a dysfunctional enzyme leads to the accumulation of heparan sulfate, especially in the liver and kidney, and to the vacuolisation of cells, including macrophages, epithelial cells and neurons^15^. Previous studies on mouse models have shown that a defect in lysosomal functions is associated with a profound adipose deficiency, and in line with this, the *Naglu* knockout mice had significantly decreased fat mass and increased lean mass, as analysed by Woloszynek *et al*.^17^ and by us in the present study. The liver was also affected, with increased weight associated with lipid and GAG accumulation, although glucose tolerance was found to be improved. Our observations on the metabolic phenotype of Hsd17b1-LacZ/Neo and Hsd17b1-LacZ/Neo X HSD17B1TG mice document a high degree of similarity with that found in the Naglu-Neo mice, except for a more severe lipid accumulation in the liver of the Hsd17b1-LacZ/Neo mice. The very similar metabolic phenotypes strongly indicate that they are mainly caused by the defect in the *Naglu* gene in all the mutant mouse lines, thus being an off-target effect in the Hsd17b1-LacZ/Neo mice. The minor differences in the metabolic phenotypes between the *Hsd17b1* mutant mouse lines are likely related to the differences in the genetic background of the mice. However, the fertility of the Naglu-Neo females was normal, indicating that the subfertility of the Hsd17b1-LacZ/Neo mice is caused by the observed lack of HSD17B1 activity rather than due to the reduced expression of *Naglu*, although the lysosomal accumulation appeared in the ovaries of both knockout mouse models.

Notably, *Hsd17b1* and *Naglu* do not show similar expression patterns in mice. While *Hsd17b1* is highly specific in the gonads^11,12,27^, *Naglu* is expressed broadly in most tissues (present study). Thus, it is interesting to note that deleting the *Hsd17b1* allele results in a severe downregulation of the *Naglu* gene also in tissues not expressing *Hsd17b1*, while disrupting the 3′-end of the *Naglu* gene does not interfere with *Hsd17b1* expression. The regulatory regions for the *Hsd17b1* and *Naglu* gene in mice have not been characterised in detail. However, in rodent ovaries, *Hsd17b1* is known to be regulated by cAMP-mediated pathways and TGF-beta family members^28,29^, although the genomic regions mediating these effects have not been determined. Interestingly, the corresponding genomic regions coding for mouse and human HSD17B1 differ in their structure. The sequence between the *HSD17B1* and *NAGLU* genes in humans extends up to 8.6 kb, including an untranscribed pseudogene of *HSD17B1* ^30,31^. It is well possible that the observed linkage between the two genes in mice is associated with the duplication of *HSD17B1* gene in humans. However, it might be of interest to search for mutations also in the 3′-non-coding region of *NAGLU*, including the genomic regions coding for *HSD17B1* and pseudogene.

In summary, replacing the *Hsd17b1* gene using a reporter cassette results in globally reduced expression of the neighbouring *Naglu* gene in all tissues. This off-target effect is likely responsible for the metabolic phenotype present in the Hsd17b1-LacZ/Neo mice described in the present study and on the International Knockout Mouse Consortium website (www.mousephenotype.org). The downregulation of *Naglu* was specific, as the expression of other genes in the genomic region was not similarly altered. Thus, the data suggest that the genomic locus of *Hsd17b1* includes a strong regulatory region that specifically affects the *Naglu* expression, or that replacing the *Hsd17b1* gene with the 4.3 kb long expression cassette with *lacZ* impacts on the chromatin conformation-dependent *Naglu* gene expression. Other recent findings have identified various milder off-target effects within 500 kb long region surrounding the targeted locus^5^. Along with our data, this indicates the need to carefully characterise the effect of gene targeting on the expression of the surrounding genomic landscape.

## Material and Methods

### Genetically modified mouse models applied

We have previously described the generation, maintenance, and genotyping of the Hsd171b-LacZ/Neo mice used in this study^13^. In short, Hsd17b1^tm1(KOMP)Vlcg^ (referred to as Hsd17b1-LacZ/Neo in this publication) ES cells were obtained from the Knockout Mouse Project (KOMP) Repository (http://www.komp.org/). In the targeted allele, the entire coding region of the *Hsd17b1* gene was replaced with a targeting vector, which included a *lacZ* reporter gene (inserted in the translation initiation site) followed by *UBC* promoter driven cDNA (*neo*) conferring neomycin resistance. In addition, Hsd17b1-LacZ mice were produced by crossing the Hsd17b1-LacZ/Neo mice with mice expressing *Cre* recombinase globally under the *Rosa26* promoter^32^, removing the *neo* gene from the mutated *Hsd17b1* locus. Furthermore, we established a mouse line by crossing the Hsd17b1-LacZ/Neo mice with transgenic mice constitutively expressing human HSD17B1 (Hsd17b1-LacZ/Neo X HSD17B1TG)^16^. Moreover, mice with a disrupted *Naglu* gene were studied (Naglu-Neo). The Naglu-Neo mice were obtained from The Jackson Laboratory (Bar Harbor, ME, USA). In these mice, exon 6 was replaced with a cassette expressing the *neo* gene under the control of the phosphoglycerate kinase (*Pgk*) promoter^15^. The structure of the *Naglu* and *Hsd17b1* loci in the different mutant mice used are described in Fig. 1. The different mouse models were screened by PCR using primer pairs for detecting the presence of the targeted and wild type (WT) alleles (primer sequences in Supplemental Table 2).

Mice were housed under controlled environmental conditions (12 hours light/12 hours darkness, at 21 ± 3 °C and humidity 55 ± 15%) at the Central Animal Laboratory of the University of Turku. For the RNA and histological analysis, mice were sacrificed using CO_2_ asphyxiation, followed by blood withdrawal from the heart before cervical dislocation. Tissues were weighed and snap-frozen in liquid nitrogen or fixed for histological analyses. All the animal experiments were approved by the Finnish Animal Ethics Committee, which fully met the requirements defined in the U.S. National Institutes of Health guidelines on animal experimentation.

### Expression of the Naglu-Hsd17b1 genomic locus *in vitro*

To determine whether the deletion of the *Hsd17b1* gene affects *Naglu* expression *in vitro*, we generated two expression constructs in pACYC177 plasmid backbone (New England Biolabs, Ipswich, MA, USA) of this genomic region with and without the genomic fragment spanning the 1.8 kb coding region of *Hsd17b1* (Fig. 1F and G). The constructs were co-transfected with a luciferase-expressing reporter plasmid (pRL Renilla luciferase reporter vector, Promega, Madison, WI, USA) into COS cells using TurboFect kit (Thermo Fisher Scientific, Wilmington, DE, USA) according to the instructions supplied by the manufacturer. Thereafter, the expression of *Naglu* in the transfected cells was analysed by qRT-PCR and normalised to *luciferase* expression (n = 6).

### Studying the metabolic phenotype

The weight of the Hsd17b1-LacZ/Neo (n = 10–11), Naglu-Neo (9–10), and Hsd17b1-LacZ/Neo X HSD17B1TG (n = 6) male mice and their WT littermates was measured weekly for three months starting at the age of eight weeks. Mice were given water and soy-free natural ingredient food (Special Diets Services, Witham, UK) *ad libitum*. The body composition, including the fat and lean mass, was measured once every two weeks by quantitative nuclear magnetic resonance (NMR) scanning (EchoMRI-700, Echo Medical Systems, Houston, Texas, USA). Glucose tolerance tests for the *Hsd17b1-LacZ/Neo* and *Naglu-Neo* mice were performed at the age of three months (n = 9–10). Prior to testing, mice were fasted for four hours, and the basal blood glucose level was measured. Glucose (1 g/kg mouse weight) was injected intraperitoneally (i.p.), and glucose was measured using FreeStyle Lite (Abbot Diabetes Care Inc., USA) from a blood sample obtained from the saphenous vein 20, 40, 60 and 90 minutes after the glucose injection.

### RNA isolation, cDNA synthesis and quantitative real-time PCR

Tissues were snap-frozen in liquid nitrogen, and the RNA extraction was carried out with Trisure (Bioline Inc., Tauton, MA, USA) according to manufacturer’s instructions. The extracted total RNA was dissolved in nuclease free water. The quantity and quality were determined with a NanoDrop ND-1000 spectrophotometer (Thermo Fisher Scientific, Wilmington, DE, USA) and agarose gel electrophoresis. The RNA was treated with DNase I (Invitrogen™, Life Technologies, Paisley, UK) or with DNase I (Sigma, Saint Louis, MO, USA), and cDNA synthesis was carried out with SensiFAST^™^ cDNA Synthesis kit (Bioline Inc., Tauton, MA, USA). Quantitative real-time PCR was performed using SYBR Green (Finnzymes, Thermo Scientific, Wilmington, DE, USA) and CFX96 real-time PCR detection system (Bio-Rad, Hercules, CA, USA). The mRNA expression of *Hsd17b1* and *Naglu* was normalised to the expression of the ribosomal protein L19 (*Rpl19*), and the expression of mRNA was quantified using the PfaffI method^33^. The sequences of the primers used for *Hsd17b1* were 5′-TTGTTTGGGCCGCTAGAAG-3′ and 5′-CACCCACAGCGTTCAATTCA-3′; for *Naglu*, 5′TCCAACAGCACGAGTTTGAG3′ and 5′CTGCGATGGCTAATCTGTCA-3′; and for *luciferase*, 5′-GATCAAAGCAATAGTTCACG-3′ and 5′-ATTTTTGATGGCAACATGGT-3′). The expression of *Hsd17b1* was analysed in the ovaries of the Naglu-Neo mice (n = 6), and the expression of *Naglu* was analysed in various tissues in the Hsd17b1-LacZ/Neo, Hsd17b1-LacZ, Hsd17b1-LacZ/Neo X HSD17B1TG mice at 3 months of age (n = 3) and in cells transfected with the BAC constructs (n = 6).

### RNA sequencing

RNA sequencing of the ovaries and male gonadal fat of the Hsd17b1-LacZ/Neo and WT mice was carried out at the Finnish DNA Microarray and Sequencing Centre (Turku Centre for Biotechnology, the University of Turku). RNA from the gonadal fat was isolated from 6 WT and 6 Hsd17b1-LacZ/Neo males, while the ovarian RNA was isolated from 6 pseudopregnant Hsd17b1-LacZ/Neo and 6 pseudopregnant WT females. The RNA quality was analysed with Agilent 2100 Bioanalyzer (Agilent Technologies, Santa Clara, CA, USA). The samples were sequenced using the HiSeq. 2500 instrument (Illumina, San Diego, CA, USA), according to protocols provided by the manufacturer, using single-end sequencing chemistry with a 50 bp read length. The reads were aligned to the mouse reference genome version mm10 assembly available at UCSC (downloaded from Illumina iGenomes website (http://support.illumina.com/ sequencing/sequencing_software/igenome.html)) using TopHat version 2.0.10^34^. HTSeq tool v.0.5.4p3 was used to count the uniquely mapped reads associated with each RefSeq annotated gene. The data were normalised using the Trimmed Mean of M-values (TMM) approach from the edgeR package, and statistical testing between sample groups (Hsd17b1-LacZ/Neo vs. WT) was carried out using the Limma package^35,36^. The cutoff for differential expression was set at a false discovery rate (FDR) < 0.01, absolute fold change above 2.0 and mean RPKM expression level > 0.125 in at least one of the groups.

### Analysis of glycosaminoglycans

The concentration of GAGs was measured according to a previously published method^37^. In brief, the snap-frozen samples (n = 3) were incubated in 0.9% NaCl, 0.2% Triton X-100 overnight at +4 °C with shaking and then homogenised with a TissueLyser (Qiagen, Hilden, Germany) using 7 mm metal beads. After homogenisation, the samples were centrifuged at 5000 g for 10 min at +4 °C, and the supernatant was collected for glycosaminoglycan quantitation. For this, the samples were mixed with 8 M guanidine-HCl and incubated for 15 min at room temperature (RT). A solution containing 54 mM sulfuric acid and 0.75% Triton X-100 was applied to the samples and mixed thoroughly. A solution of Alcian blue (Sigma-Aldrich, Saint Louis, MO, USA), 1.8 M sulfuric acid, 8 M guanidine-HCl and 10% Triton X-100 was added and the samples were incubated for 1 hour at +4 °C. Next, the samples were centrifuged at 12 000 g for 15 min, and the supernatant was discarded. The pellet was washed with 40% DMSO, 50 mM MgCl_2_ solution, mixed with 4 M guanidine-HCl, 33% isopropanol and agitated at RT for 15 min. The absorbances of samples were then determined at a wavelength of 600 nm.

### Histology and immunohistochemistry

For histological analysis, tissues were fixed in 10% buffered formalin solution at RT for 24 h. After fixation, tissues were dehydrated and embedded in paraffin. Five µm thick sections were prepared, deparaffinised and rehydrated, and stained with haematoxylin and eosin. In addition, Oil Red O staining was performed on 10 μm thick cryosections according to standard procedures. Briefly, sections were fixed in buffered formalin solution followed by washing with water and isopropanol. The sections were stained in Oil Red O solution and then washed with isopropanol and water, followed by mounting with Aquatex® (Merck, Darmstadt, Germany).

For immunohistochemistry, formalin-fixed and paraffin-embedded samples were sectioned prior to deparaffinisation and rehydration. Five μm thick sections were exposed to antigen retrieval in a pressure cooker with sodium citrate buffer for 30 minutes. The blocking reaction with 10% goat serum and 3% bovine serum albumin (BSA; Sigma-Aldrich) in PBS for non-specific binding was performed, followed by overnight incubation with the primary antibody against LAMP1 (dilution 1:400, ab24170, Abcam, Cambridge, UK) or LIMP2/lpg85 (dilution 1:400, NB400-129, Novusbio, Littleton, CO, USA) and CYP17A1 (dilution 1:2000, #PTGlab14447; Proteintech Group, Chicago, IL, USA) at 4 °C. Endogenous peroxidase activity was then blocked by incubation in 3% H_2_O_2_ for 20 min at RT, followed by a 30 min incubation at RT with an anti-rabbit antibody conjugated with polymer-HRP (HRP-conjugated polymer against rabbit, Dako EnVision+ System; Dako, Carpinteria, CA, USA). The sections were then washed, and the bound antibody was visualised with Envision™+ System-HRP DAB staining (Dako, Carpinteria, CA, USA). The sections were counterstained with haematoxylin, mounted and digitised using a Pannoramic 250 slide scanner (3DHISTECH, Budapest, Hungary).

### Measurement of NAGLU activity

NAGLU activity was determined fluorometrically as previously described by Li *et al*. with minor modifications^15^. In brief, approximately 30 mg of tissue sample was homogenized in 0.9% NaCl/0.2% Triton-X 100 by hand with the pestle, rotated at +4 °C for 2 hours and centrifuged (5000 g for 10 min) to remove the debris. Supernatants were collected and 25 µl aliquots were incubated for 1 hour in +37 °C with 25 µl of 0.2 mM 4-methylumbelliferyl-2-acetamido-2-deoxy-α-D-glycopyranoside (Abcam, Cambridge, UK) in 0.1 M Na acetate buffer (pH 4.3) containing 0.5 mg/ml BSA. Reaction was terminated by adding 1 ml of 10 mM glycine buffer (pH 10.5). The 4-methylumbelliferone released was measured with fluorometer (excitation wavelength of 355 nm, emission wavelength of 460 nm, exposure time 0.1 sec; Victor x 4; PerkinElmer, Waltham, MA, USA). For determining the calibration curve, 4-methylumbelliferone (Sigma-Aldrich) was used and the NAGLU activity was determined as nmol/h/mg tissue weight.

### HSD17B activity

Kidney samples (approximately 30 mg in weight) were homogenized by incubating them in 0.9% NaCl, 0.2% Triton X-100 overnight at +4 °C with shaking and then homogenised with a TissueLyser (Qiagen, Hilden, Germany) using 7 mm metal beads. The depris was removed by centrifuging (5000 g for 10 min). Reaction mixture (200 µl) containing 20 mM KH_2_PO_4_ with 1 mM EDTA and protease inhibitor (Roche Diagnostics, Indianapolis, IN, USA), pH 7.4, 1 mM β-NADPH (Merck), 30 nM E1 (Sigma-Aldrich), and 5 nM [^3^H]-E1 (PerkinElmer) was mixed with 10 µl sample homogenate and incubated for 2 hours at RT. Reaction was stopped with 6.1 N trichloroacetic acid (Sigma-Aldrich, final concentration 1% v/v). Samples were the applied to Waters Ostro sample preparation plates (Waters Co., Milford, MA, USA) and the steroids were eluted from the plate with 100 µl acetonitrile followed with 100 µl of MilliQ-water. The amount of [^3^H]-E1 and [^3^H]-E2 in the samples was analyzed with HPLC connected to on-line with a scintillation analyser. As HPLC we used Waters Acquity UPLC H-class (Waters Co.) equipped with XBridge C18 (3.5 µm, 4.6 × 50 mm; Waters Co.) column and XBridge VanGuard C18 (3.5 µm, 3.9 × 5 mm; Waters Co.) guard column. As mobile phase we used acetonitrile (VWR) – water (48/52 v/v) with flow of 1.2 ml/min. The eluent was mixed on-line with scintillant (Ecoscint A, National Diagnostics, Atlanta, GA, USA) with flow of 0,6 ml/min, and beta emission of the [^3^H]-steroids was counted on-line with Berthold FlowStar^2^ (Berthold Technologies GmbH, Bad Wildbad, Germany) scintillation analyzer. The HSD17B activity was calculated based on the percentage of [^3^H]-E1 converted to [^3^H]-E2, and determined as pg of E2 formed/ug protein.

### Statistical analyses

Statistical analyses, except for the RNA sequencing data, were carried out using GraphPad Prism 7 software (GraphPad Software, Inc., La Jolla, CA, USA). Statistical significance between groups, which was set at P < 0.05, was determined using an unpaired t-test or 2-way ANOVA followed with Bonferroni *post hoc* test, and mean values ± SD or SEMs are presented.

### Data availability statement

All data generated or analysed during this study are included in this published article (and its Supplementary Information files) or can be requested from the corresbonding author.

## Electronic supplementary material

Supplementary information
