# Supplementary material for: Deleting the mouse Hsd17b1 gene results in a hypomorphic Naglu allele and a phenotype mimicking a lysosomal storage disease
Source: Sci Rep. 2017 Nov 27;7:16406. doi: 10.1038/s41598-017-16618-5 (PMC5703720; doi:10.1038/s41598-017-16618-5)
Supplement: Supplementary file 1 — Supplementary information [file 41598_2017_16618_MOESM1_ESM.pdf]

Supplementary information

Title

Deleting the mouse *Hsd17b1* gene results in a hypomorphic *Naglu* allele and a phenotype mimicking a lysosomal storage disease

Authors

Heli Jokela, Janne Hakkarainen, Laura Kätkänaho, Pirjo Pakarinen, Suvi T. Ruohonen, Manuel Tena-Sempere, Fu-Ping Zhang, Matti Poutanen

Supplementary Figure S1. *Naglu* expression, GAG and histological and immunohistochemical analyses of female mice. Quantitative RT-PCR (A) analysis of the ovary, uterus, kidney and liver showed the downregulation of *Naglu* expression in the Hsd17b1-LacZ/Neo female mice (white bars) and Hsd17b1-LacZ mice (dark grey bars) compared with WT female mice (black bars) at 3 months of age. Bars demonstrate the mean of the relative expression normalised to the expression of *L19* (the error bars show SD, n=6). (B) Alcian blue precipitable GAG (mean ± SEM) was analysed from the tissues of Naglu-Neo, Hsd17b1-LacZ/Neo, Hsd17b1-LacZ and Hsd17b1-LacZ/Neo X HSD17B1TG female mice and their control littermates (n=3). Immunohistochemical staining for LAMP1 was used to demonstrate lysosomes in the ovaries and uterus of Hsd17b1-LacZ/Neo female mice. Increased staining was observed in the uterus (D) and mild staining in the ovary (F). LAMP1 immunohistochemistry marked the areas of large vacuoles within the uterus of the Hsd17b1-LacZ/Neo mice (H). Histological sections stained with HE showed vacuolisation in the uterus and ovary of Hsd17b1-LacZ/Neo and Naglu-Neo mice (K, L, M and N).

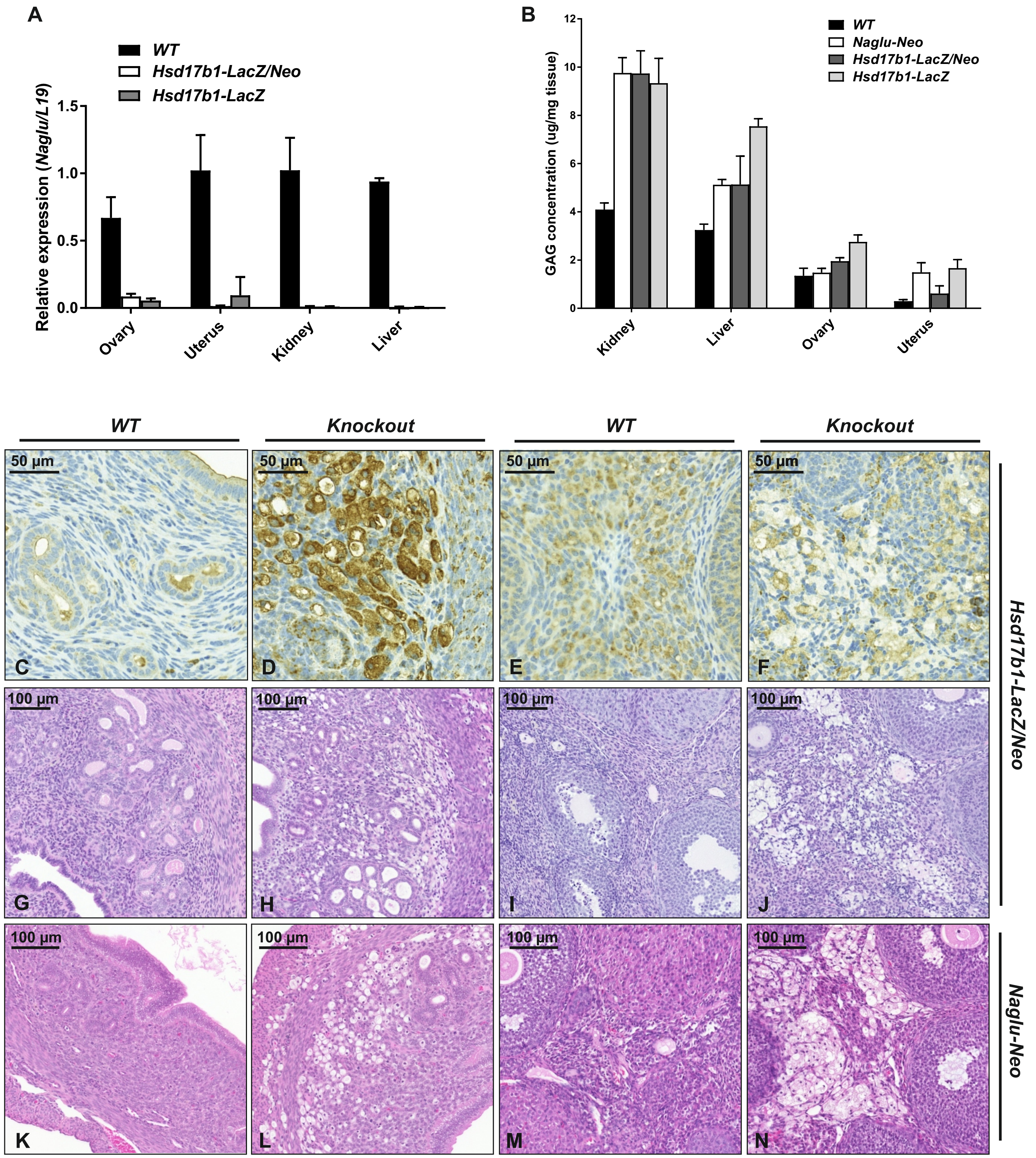
A
WT
Hsd17b1-LacZ/Neo
Hsd17b1-LacZ
Relative expression (Naglu/L19)
1.5
1.0
0.5
0.0
Ovary
Uterus
Kidney
Liver
B
GAG concentration (ug/mg tissue)
12
10
8
6
4
2
0
WT
Naglu-Neo
Hsd17b1-LacZ/Neo
Hsd17b1-LacZ
Kidney
Liver
Ovary
Uterus
WT
Knockout
WT
Knockout
50 µm
100 µm
C
D
E
F
G
H
I
J
K
L
M
N
Hsd17b1-LacZ/Neo
Naglu-Neo

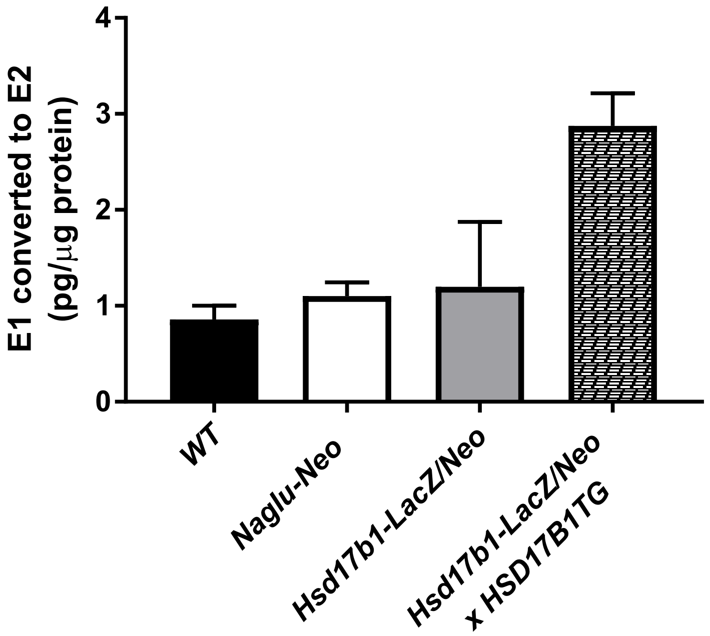

Supplementary Figure S2. HSD17B activity measured in kidney homogenates of mice with different genotypes. The data indicate markedly increased HSD17B activity in Hsd17b1-LacZ/Neo X HSD17B1TG mice compared with WT, Naglu-Neo and Hsd17b1-LacZ/Neo mice. Bars demonstrate the mean of the relative expression normalised to the expression of *L19* (the error bars show SD, n=6).

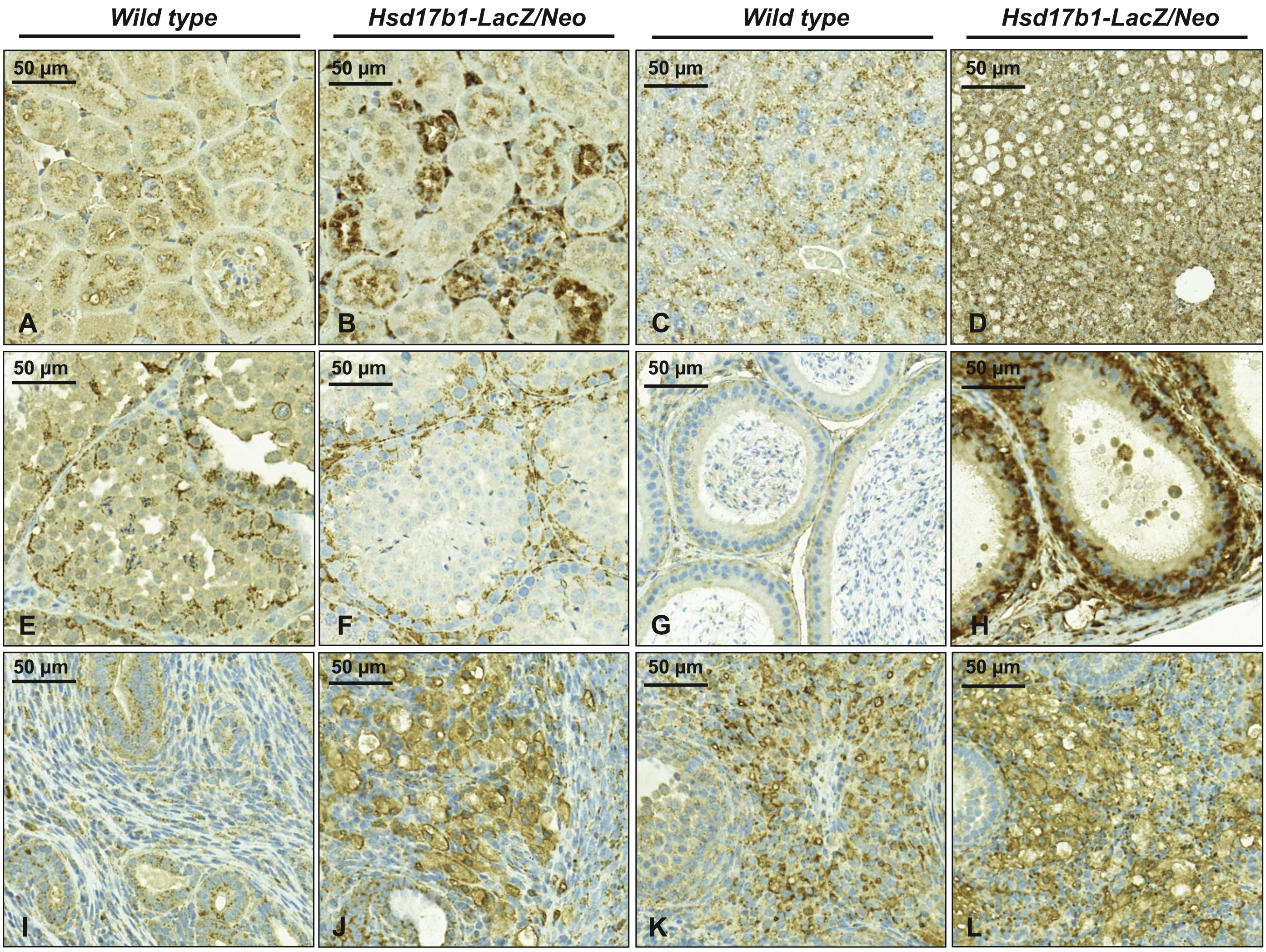

Supplementary Figure S3. Immunohistochemical analysis of Hsd17b1-LacZ/Neo mice. Immunohistochemical staining for LIMP2 was used to demonstrate lysosomes in Hsd17b1-LacZ/Neo mice. Increased staining was observed in kidney (B), liver (D), epididymis (H), uterus (J) and ovary (L) while no difference in the intensity of staining was shown in testis sections from WT and Hsd17b1-LacZ/Neo mice (E and F).

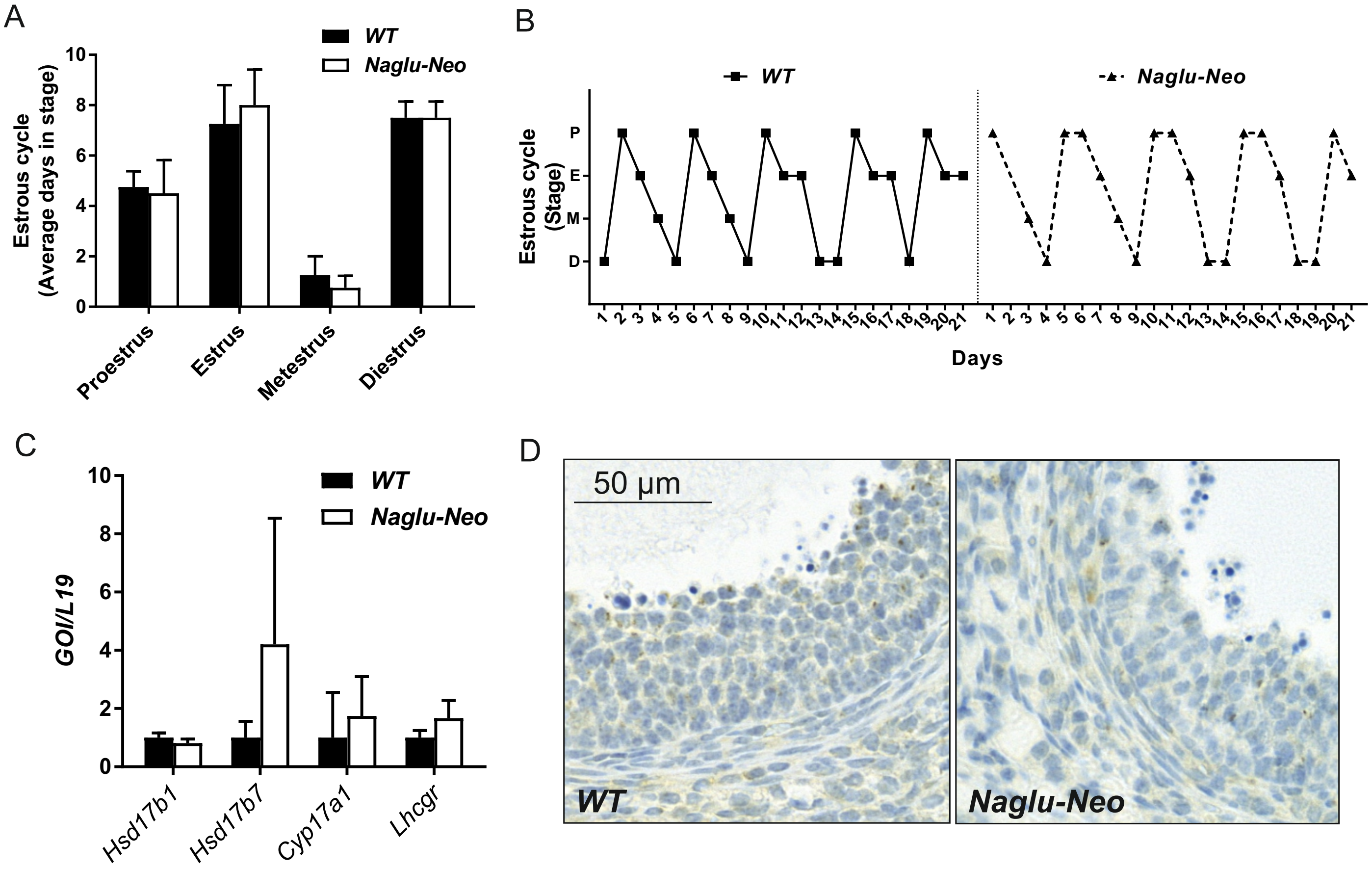

E

Genotype	No. of plugs/female	No. of litters/female	Plugs/ litter	Litter size
WT	4.5	1.8	2.6	7.0
Naglu-Neo	5.3	2.4	2.2	7.1

Supplementary Figure S4. Fertility of Naglu-Neo female mice. (A) Time spent in different phases of estrous cycle during a 21 d follow-up period was similar with Naglu-Neo mice compared to WT littermates. (B) Representative estrous cycle of the WT and Naglu-Neo female mice throughout the 21 d follow-up period. P, proestrus; E, estrus; M, metestrus; D, diestrus. Estrous cycle was determined by vaginal cytology method. (C) The mRNA expression of genes, *Hsd17b1*, *Hsd17b7*, *Cyp17a1* and *Lhcgr*, that were altered in Hsd17b1-LacZ/Neo mice, were not changed significantly in pseudopregnant ovaries of Naglu-Neo female mice, (D) nor was the immunohistochemical staining intensity for CYP17A1 different between WT and Naglu-Neo mice. (E) When Naglu-Neo and control littermate female mice were mated with WT male mice of proven fertility for a 60 d period, it became evident that the female mice present with normal fertility and litter size.

Supplementary Table S1. RNAseq data of the gene expression of genes locating at the vicinity of mouse *Hsd17b1* gene (+/- 500 kb). Numbers for the KO (Hsd17b1-LacZ/Neo mice) and WT (wild type mice) columns indicate mean RPKM expression values. FC = fold change, gWAT =gonadal white adipose tissue.

		Fold changes in RNAseq								
		FC		Mean expression		FC		Mean expression		
	Gene ID	Ovary	P-value	KO	WT	gWAT	P-value	KO	WT
	Dnajc7	1,08	NS	104,72	100,92	1,09	NS	79,33	72,79
	Nkiras2	-1,08	NS	118,17	127,93	-1,12	NS	127,27	142,65
	Zfp385c	-1,51	NS	0,03	0,03	1,96	NS	0,12	0,06
	Hspb9	1,84	NS	0,16	0,08	3,83	NS	0,41	0,11
	Rab5c	1,10	NS	69,03	62,61	-1,18	NS	134,47	158,27
	Kcnh4	2,06	NS	0,13	0,07	-1,28	NS	0,37	0,48
	Kat2a	1,08	NS	91,53	85,01	-1,01	NS	19,84	19,95
	Ghdc	-1,05	NS	4,48	4,70	-1,12	NS	9,66	10,84
	Hcrt	2,05	NS	0,02	0,01	-1,14	NS	0,13	0,15
	Stat5b	1,05	NS	106,29	101,26	-1,12	NS	24,88	27,84
	Stat3	1,15	NS	59,29	51,63	-1,02	NS	44,64	45,44
	Ptrf	-1,19	NS	27,26	32,54	-1,47	NS	1161,10	1711,02
	Atp6v0a1	-1,29	0.017	111,98	144,34	-1,26	0.04	29,68	37,49
Hsd17b1	*Naglu*	-29,20	1.8e-11	1,33	38,82	-40,51	4.3e-7	1,07	43,47
	Coasy	1,06	NS	28,23	26,57	1,39	0.02	67,90	48,76
	Mlx	1,51	0.003	71,77	108,00	-1,61	NS	44,95	72,23
	Fam134c	-1,11	NS	147,67	163,26	-1,18	0.02	46,73	55,00
	Psmc3ip	-1,37	NS	0,57	0,78	1,07	NS	5,35	5,31
	Tubg1	-1,02	NS	11,15	11,40	-1,59	0.02	5,13	8,14
	Tubg2	1,18	NS	4,74	4,03	-1,59	0.04	0,35	0,55
	Plekhh3	-1,24	NS	46,65	57,94	-1,27	NS	4,31	5,45
	Ccr10	-3,11	0.00051	0,19	0,59	-1,12	NS	0,19	0,21
	Cntnap1	-2,60	2.5e-7	1,81	4,72	-2,06	0.01	8,10	16,72
	Ezh1	-1,20	0.0285	129,26	154,88	-1,15	NS	13,74	15,72
	Ramp2	-1,08	NS	16,53	16,84	1,66	NS	10,56	14,89
	Wnk4	-1,32	NS	1,54	2,03	1,41	NS	9,09	3,78
	Cntd1	-1,05	NS	1,50	1,10	1,43	NS	0,39	0,27
	Becn1	1,02	NS	34,13	33,56	-1,11	NS	8,96	9,96
	Vps25	-1,07	NS	40,82	43,79	-1,24	NS	43,25	53,55
	Psme3	-1,01	NS	115,81	116,88	-1,12	NS	25,03	28,20
	Aoc2	1,08	NS	4,90	4,52	1,25	NS	4,35	3,49
	Aoc3	1,49	NS	84,22	56,55	-1,45	0.03	1437,04	2086,25
	G6pc	2,29	NS	0,17	0,07	1,01	NS	0,26	0,26
	Rundc1	-1,03	NS	2,79	2,86	1,04	NS	10,98	10,52
	Aarsd1	1,04	NS	22,16	21,30	-1,17	NS	21,18	24,87
	Rnd2	-1,64	0.0212	9,89	16,19	-1,48	NS	11,12	16,49
	Vat1	-1,03	NS	52,08	53,40	1,27	0.02	95,73	75,15
	Ifi35	-1,14	NS	4,44	5,04	-1,23	NS	14,93	18,41
	Rpl27	-1,15	NS	11,73	10,70	1,14	NS	0,94	0,82
	Brca1	-1,02	NS	282,74	287,31	-1,23	NS	0,47	0,58
	Nbr1	-1,15	NS	49,83	56,01	-1,03	NS	89,04	83,10

Supplementary Table S2. Genotyping primers for Hsd17b1-LacZ/Neo, Hsd17b1-LacZ, HSD17B1TG and Naglu-Neo mice

Mouse model	Allele	Forward primer sequence	Reverse primer sequence
**Hsd17b1-LacZ/Neo**	WT	SU: 5'-CAACCCATCCCTTGATCAAC-3'	exon1as1: 5'-CATCATCGTGCTTACCTCTG-3'
	Targeted	SU: 5'-CAACCCATCCCTTGATCAAC-3'	LacZR: 5'-GTCTGTCCTAGCTTCCTCACTG-3'
**Hsd17b1- LacZ**	Targeted	SV40: 5’-GTTTATTGCAGCTTATAATG-3’	SD: 5’-AACCCTTAGCCTAGCACCTTG-3’
**HSD17B1TG**	Targeted	Fw2: 5’-CTTCAGATCCATCCCAGAGC-3’	Ex32: 5’-GCCCAGGCCTGCGTTACAC-3’
**Naglu-Neo**	WT	NagluF: 5’-GTCGTCTCCTGGTTCTGGAG-3’	NagluWTR: 5’-ACCACTTCATTCTGGCCAAT-3’
	Targeted	NagluF: 5’-GTCGTCTCCTGGTTCTGGAG-3’	NagluKOR: 5’-TGGATGTGGAATGTGTGCGAG-3’
